# A review on deep learning methods for heart sound signal analysis

**DOI:** 10.3389/frai.2024.1434022

**Published:** 2024-11-13

**Authors:** Elaheh Partovi, Ankica Babic, Arash Gharehbaghi

**Affiliations:** ^1^Department of Electrical Engineering, Amirkabir University of Technology, Tehran, Iran; ^2^Department of Biomedical Engineering, Linköping University, Linköping, Sweden; ^3^Department of Information Science and Media Studies, University of Bergen, Bergen, Norway

**Keywords:** phonocardiogram, intelligent phonocardiography, deep learning, heart sound, heart sound segmentation, heart disease, end-to-end learning, heart sound classification

## Abstract

**Introduction:**

Application of Deep Learning (DL) methods is being increasingly appreciated by researchers from the biomedical engineering domain in which heart sound analysis is an important topic of study. Diversity in methodology, results, and complexity causes uncertainties in obtaining a realistic picture of the methodological performance from the reported methods.

**Methods:**

This survey paper provides the results of a broad retrospective study on the recent advances in heart sound analysis using DL methods. Representation of the results is performed according to both methodological and applicative taxonomies. The study method covers a wide span of related keywords using well-known search engines. Implementation of the observed methods along with the related results is pervasively represented and compared.

**Results and discussion:**

It is observed that convolutional neural networks and recurrent neural networks are the most commonly used ones for discriminating abnormal heart sounds and localization of heart sounds with 67.97% and 33.33% of the related papers, respectively. The convolutional neural network and the autoencoder network show a perfect accuracy of 100% in the case studies on the classification of abnormal from normal heart sounds. Nevertheless, this superiority against other methods with lower accuracy is not conclusive due to the inconsistency in evaluation.

## 1 Introduction

The context of biomedical engineering has been considerably enhanced after the development of Artificial Intelligence (AI) and Deep Learning (DL) methods. This enhancement can be profoundly seen in different applications of AI-based methods including automated cardiac disease diagnosis using a recording of heart sound signal, called Phonocardiograph (PCG), as the input to the DL method. This domain of computing methods has addressed various embodiment, from the traditional AI-based methods (Sepehri et al., 2008), to the hybrid models (Gharehbaghi et al., 2015a,b), and ultimately DL methods, over the previous decades (Gharehbaghi et al., 2019b, 2017b). The shift from the traditional to the hybrid methods was not indeed as effective as the leap from the hybrid to the DL method. Although Artificial Neural Networks (ANN) first emerged as an alternative to the statistical methods, i.e., Hidden Markov Model (HMM), the theoretical link between these two alternatives was later understood by the researchers (Bourlard and Wellekens, 1990). Regardless of using ANN or HMM, feature extraction is a step with fundamental importance, which has always tried to be elaborated to secure an acceptable performance of the learning method. On the contrary, a DL method can be designed in a manner to learn appropriate features for reliable performance. Such advancement was not seen in the former alternatives.

Application of DL methods has been expanded to PCG analysis mainly in two different ways: classification of abnormal heart conditions from the normal ones, and segmentation of PCG signal, where the latter implies the process by which onset and endpoint of the basic heart sounds are identified. It is worth noting that a heart creates two sounds, resulting from the valvular closure, named the first and second heart sound. These two sounds are known as the basic heart sounds and the time intervals between basic heart sounds carry important information about heart condition. The importance of an expert system for cardiac disease diagnosis is better understood if considering that cardiovascular disease is still the main cause of human mortality.

Several architectures of deep learning methods have been introduced for PCG analysis, either for classification or for segmentation purposes, however, result discrepancy along with the inconsistent training/validation circumstances make selection of a reliable DL method a complicated task.

To put this point into a better perspective a number of the challenges faced with the development of an expert system for cardiac disease diagnosis based on PCG analysis, are listed as follows:

- PCG signal by itself, is non-stationary, nonergodic, and cyclic signal (Gharehbaghi et al., 2019b).
- A recorded PCG may contain different contaminating sources such as noises and artifacts (Deperlioglu et al., 2020; Baghel et al., 2020).
- The frequency characteristics of the stethoscope can make models to be biased toward the majority sources of training data (Humayun et al., 2020).

It is, therefore, unrealistic to rely on the performance measures of the DL methods without considering the training/validation dataset. Such technical details can not be found in some of the review papers, in which the application of DL methods in cardiac disease diagnosis was highlighted (Fernando et al., 2021; Lakshmi et al., 2021; Abdullah Aloyuni, 2021). A number of review papers reported the power of deep learning methods for PCG classification (Bizopoulos and Koutsouris, 2018; Chen W. et al., 2021; Li S. et al., 2020). However, all the studies fail to provide sufficient details, in terms of the taxonomy as well as the computational methodology, for the researchers and engineers to select appropriate methods for their research objectives. For example, a great majority of the DL methods are applied to certain segments of PCG. This necessitates another computational step, named the segmentation process, since manual segmentation makes the method accurate, and user-dependent. Such computational details cannot be found in the review papers.

This paper represents the results of a comprehensive study on DL methods that were employed for PCG analysis. The main objective of the paper is to provide an overview of different DL methods along with their applicability, restrictions, and criteria, in light of PCG processing. The methodological taxonomy is based on the processing objectives, e.g. classification and segmentation. Results of each DL method in conjunction with the corresponding training/validation dataset are represented for each study objective separately. In addition to introducing technical contents of the common DL methods used for PCG processing, detailed results will be represented in tabular form to be used as a quick reference, categorized based on the DL focus. Moreover, the complexity of the DL methods as well as the corresponding performance will be described separately.

The main contributions of the paper are:

- Introducing a novel taxonomy for heart sound analysis based on the applicability of the published studies and performing a pervasive review of the studies according to the introduced taxonomy. The introduced taxonomy can help the researchers and developers to find the existing methodologies that suit their research questions.
- Presentation of the trend of the various DL methods for heart sound analysis using a new representation. This can help the researchers to understand the progress of various DL methods in the domain.
- Presentation of the technical details of DL methods used for PCG analysis, including the segmentation process.
- Pre metrics that exist within the related community. The existing studies have mostly overlooked the importance of the database in the learning process.
- Representing the survey results in terms of the applicative and the methodological taxonomy.
- Conclusive representation of the most popular and the most accurate DL methods based on the introduced taxonomy.

The paper provides a clear picture of the capabilities and restrictions of the DL methods to be used as a reliable computing method for PCG analysis. The DL methods are not studied based on the performance only, and the validation databases as well as the research questions are investigated. It is worth noting that DL methods are always accompanied by classification errors, and therefore, further medical measures might be eventually needed to admit the methods to the clinical settings. Nevertheless, representation of the method performance for a research question can provide a baseline for the researchers to further investigate. Moreover, the resulting computing method can be incorporated into an Internet of Things structure to serve as an easy-to-use decision support system for this demanding clinical application (Gharehbaghi and Lindén, 2015; Gharehbaghi et al., 2019c).

## 2 Medical background

**Figure 1:**
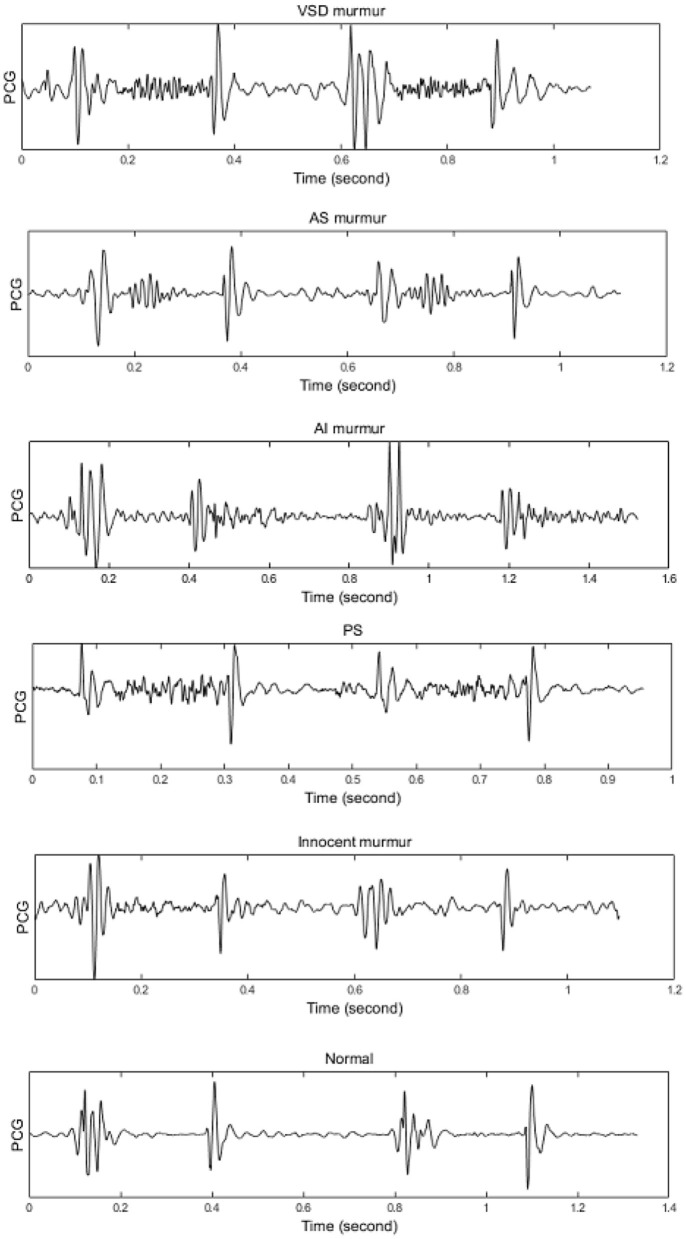
Illustrating different heart diseases including: VSD, AS, AR(AI), PS, Innocent Murmur, Normal. Source: Sepehri et al. (2008).

A heart normally encompasses 4 chambers, two smaller on the top named atrium, and the larger ones named ventricle. A wall named septum separates the two ventricles from each other, and so do the atriums, however, the left/right atrium is separated from the left/right ventricle by mitral/tricuspid valves. There are two other valves between the left/right ventricle and the artery that carries blood to the body/lungs, named the aortic pulmonary valve. The heart walls are normally contracted and relaxed rhythmically, such that more than 55% of the incoming blood is typically ejected into the aortic root. A heart that is not capable of ejecting more than 40% of the blood is evidenced as the HEART FAILURE case. A normal heart has a cyclic mechanical activity that creates an acoustical signal. A recording of this acoustical, or PCG, contains two basic sounds, named the first heart sound (S1) and the second heart sound (S2), which result from the closure of the mitral/tricuspid and aortic/pulmonary valves, respectively. conditions such as an obstructed valve, a shunt on any of the septum, or valvular leakage results in blood turbulence, named murmur. Nevertheless, a normal heart might initiate a murmur, named innocent murmur, which is mostly heard in children. Discrimination between the murmurs is a complicated task, especially considering the non-stationary and ergodic properties of the PCG signal which makes a high between-class similarity. Figure 1 demonstrates two cycles of PCG for a case with a shunt in the ventricular septum (VSD), an aortic obstruction (AS), an aortic leakage (AR), a pulmonary obstruction (PS), an innocent murmur, and a normal (no murmur) condition. The signals were selected from our previous data acquisition in compliance with the codes of the World Medical Association, whose details can be found in Sepehri et al. (2008).

## 3 Deep learning methods

### 3.1 Convolutional neural networks

**Figure 2:**
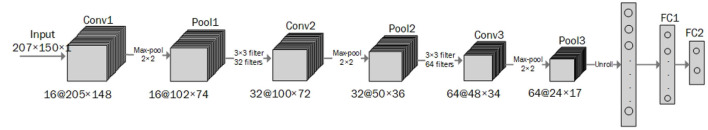
Illustration of an architecture for Convolutional Neural Network (CNN) architecture.

CNN is a deep learning method designed to process multiple arrays of data through back-propagation of the learning error using several layers such as convolution layers, batch normalization layers, pooling layers, and fully connected (FC) layers. The convolutional layers perform feature extraction by applying kernels (filters) to their inputs (LeCun et al., 2015; Meintjes et al., 2018; Yamashita et al., 2018). Two principal advantages of using a convolution layer instead of the fully connected layer are parameter sharing and sparsity of connections (Yamashita et al., 2018). The pooling layer down-samples the output of the middle layers to reduce overfitting. The max-pooling and global average pooling are considered two common operations of pooling (Meintjes et al., 2018; Renna et al., 2019; Yamashita et al., 2018). The batch normalization layer is responsible for developing a faster and more stable network through the normalization of the activation of each channel while, the fully connected layer is responsible for classification in which the output of the last convolution or pooling layer is unrolled into a vector and then connected to one or more fully connected layers (Meintjes et al., 2018; Yamashita et al., 2018). Rectified linear unit (ReLU) is an activation function used after each convolutional layer and has advantages over a sigmoid activation function in reducing the likelihood of vanishing gradient and sparsity (Maknickas and Maknickas, 2017). The activation function applied to the last fully connected layer is different for various classification problems (Yamashita et al., 2018). An architecture for the CNN used for the classification task is shown in Figure 2 (Meintjes et al., 2018). Table 1 shows a list of the learning and design parameters, commonly employed by CNNs.

**Table 1:** List of parameters and design parameters in CNN, RNN, and TGNNs.

	**Parameters**	**Design parameters**
CNN	Weights of FC layer, Weights of kernels	Number of kernels in convolutional layers, kernel size, stride, padding, activation functions, Pooling method, filter size of pooling layers, stride, padding, Number of units in FC layer, Learning rate, cost function, mini-batch size, number of epochs, and regularization
RNN	Weights	Number of neurons in the hidden layer, number of hidden layers, number of units in a dense layer, RNN model (LSTM, GRU, BiLSTM, BiGRU), activation function, dropout, decay rate, momentum, Learning rate, cost function, mini-batch size, number of epochs, regularization
TGNN	Discriminative frequency bands, growing time scheme, and learning weights	Number of neurons in the hidden layer, number of growing sectors, activation function, Learning rate, cost function, mini-batch size, number of epochs, and regularization

### 3.2 Recurrent neural networks

**Figure 3:**
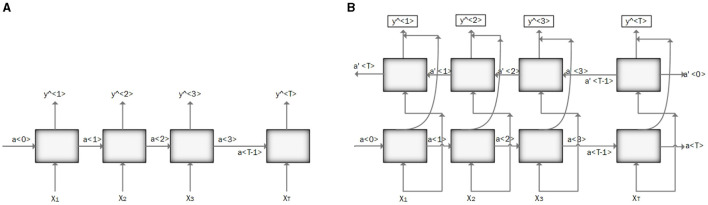
**(A)** Illustration of recurrent neural network (RNN) architecture. **(B)** Illustration of bidirectional recurrent neural network (BRNN) architecture.

RNN is designed to process sequential data and model temporal dependencies between sequential data (Messner et al., 2018). The network has an input layer with no size limit, a hidden layer (hidden state) that depends on all the previous hidden states, and an output layer. Vanishing and exploding gradients are the two major problems in RNN performance. Vanishing gradients occur when gradients become very small, leading to slow learning and poor performance on long-term dependencies. Conversely, exploding gradients happen when gradients become very large, causing to instability and failure to converge. Using gated architecture such as Long Short-Term Memory (LSTM) or Gated Recurrent Units (GRUs) can help to overcome these problems (Mikolov et al., 2010; Latif et al., 2018). Figure 3A illustrates the RNN architecture.

Bidirectional Recurrent Neural Networks (BRNNs) have been proposed to incorporate future contents of a sequential into the learning process along with the past data points (Messner et al., 2018). This change can be applied to any model that uses RNN, GRU, or LSTM. It can make predictions anywhere in the sequence by considering information from the entire sequence. Figure 3B illustrates the BRNN architecture.

**Figure 4:**
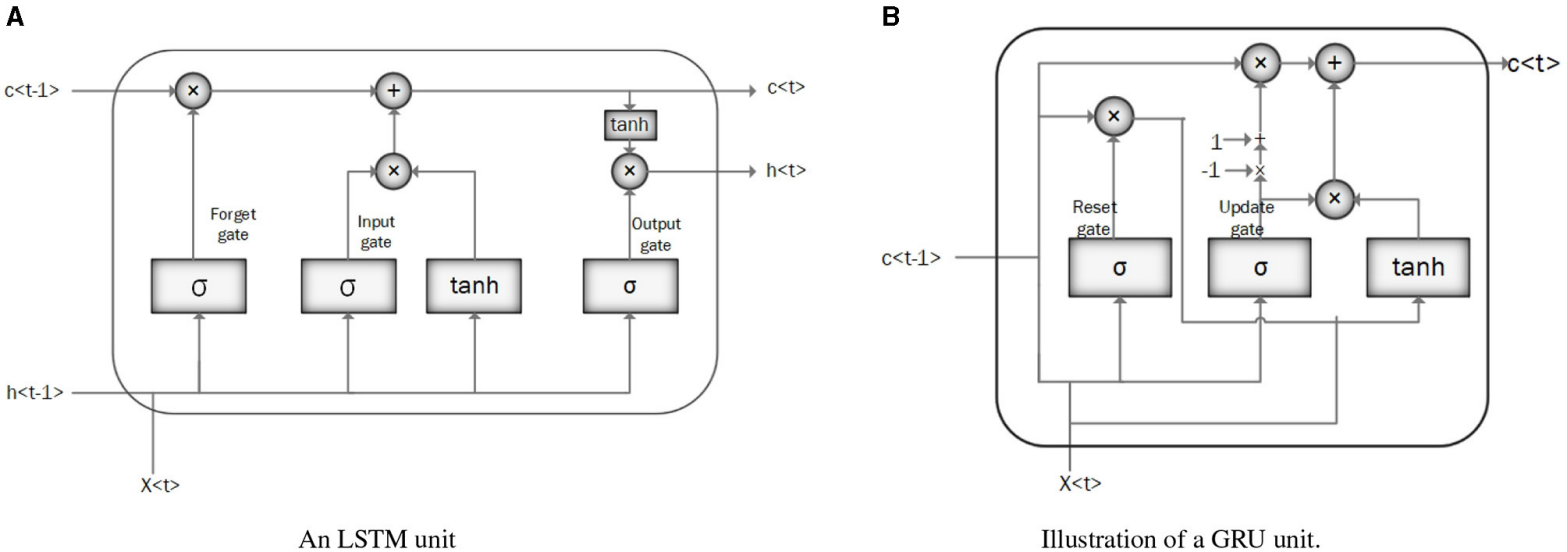
Illustration of an LSTM and GRU unit. **(A)** An LSTM unit. **(B)** Illustration of a GRU unit.

- Long Short-Term Memory (LSTM): LSTM is a modified version of RNN that resolves a common bottleneck of RNNs: vanishing and exploding gradients, by capturing long-term time dependencies (Chung et al., 2014; Latif et al., 2018). LSTM architecture consists of recurrent memory blocks. Each memory block consists of three gates, input, output, and forget gate to control its content (Messner et al., 2018). The general structure of the LSTM network is the same as in Figure 3A, except that instead of RNN units, the LSTM units. are employed. Figure 4A illustrates the LSTM unit.
- Gated Recurrent Unit (GRU): GRU is a simplified structure of LSTM with less computational cost. In GRU, the input gate and the forgetting gate, are combined to form a new gate, called the “update gate.” GRU also includes another gate called the reset gate in its structure to cope with the vanishing and exploding gradient efficiently. These gates adjust the information flow in the unit (Chung et al., 2014; Latif et al., 2018; Messner et al., 2018; Sujadevi et al., 2017). As mentioned in the previous section, the general structure of a GRU network is similar to the one in Figure 3A, except for the GRU units which are employed instead of RNN units. Figure 4B illustrates the GRU unit.

Table 1 shows a list of the learning and design parameters of an RNN.

### 3.3 Recurrent convolutional neural network

RCNN in the scope of our study refers to the combination of RNN and CNN, often a cascade of RNN and CNN, to capture long temporal context information and invariant spatial-temporal contents, respectively.

### 3.4 Time growing neural networks

**Figure 5:**
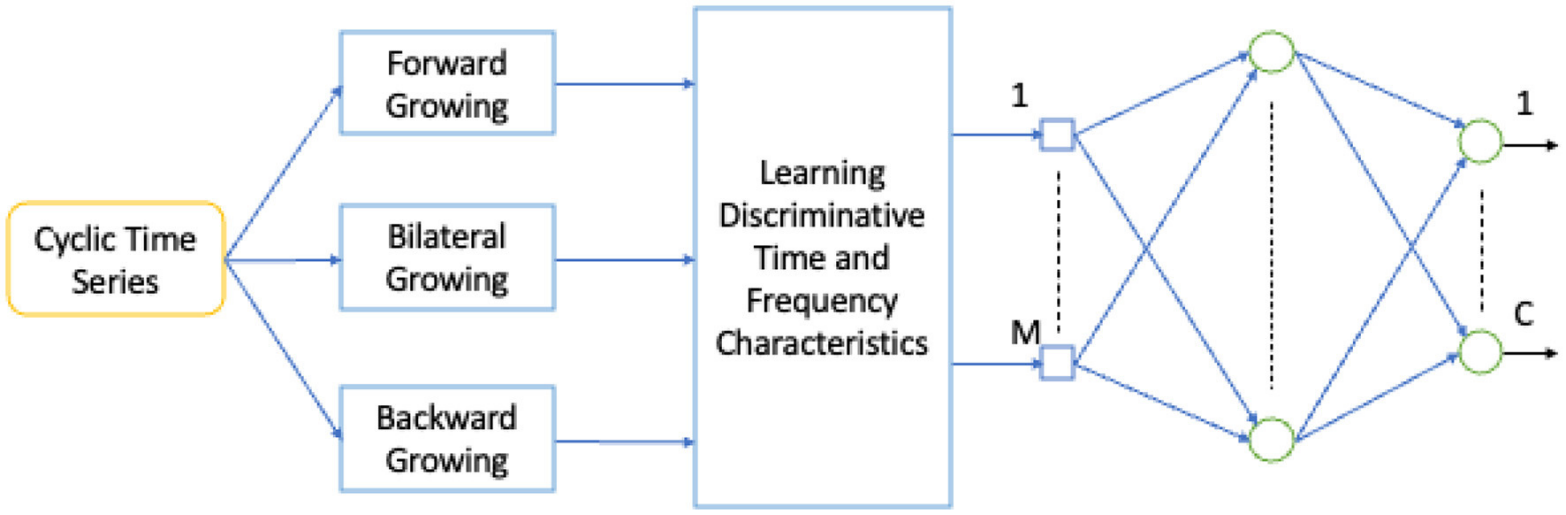
A TGNN architecture.

TGNN is a nonlinear deep learning method for learning the frequency contents of a set of temporal windows. There are three types of TGNN: forward, backward, and bilateral. In each type, a starting point is fixed and the window length grows in time until covering the entire learning segment of the signal. The use of the growing windows is specifically valuable for short-length signals, where the trade-off between temporal and spectral resolution is problematic (Gharehbaghi et al., 2014, 2015c). Deep Time Growing Neural Network (DTGNN) is an architecture of deep learning that uses TGNN units as the core of the learning process. DTGNN has three levels of learning that include between classes, over classes, and classification. A DTGNN at its deep level finds a set of discriminative frequency bands, defined as the frequency bands whose spectral contents provide an optimal separability between the classes. DTGNN introduced a way of finding the optimal discriminative frequency bands, by using the K-means method in conjunction with the Fisher criterion. Then, spectral contents of the discriminative frequency bands are considered as the input layer of the TGNN, and the training is performed using the backpropagation error method. An architecture for the TGNN used for the classification task is shown in Figure 5.

Table 1 shows a list of parameters and design parameters in TGNNs.

### 3.5 Deep belief networks

**Figure 6:**
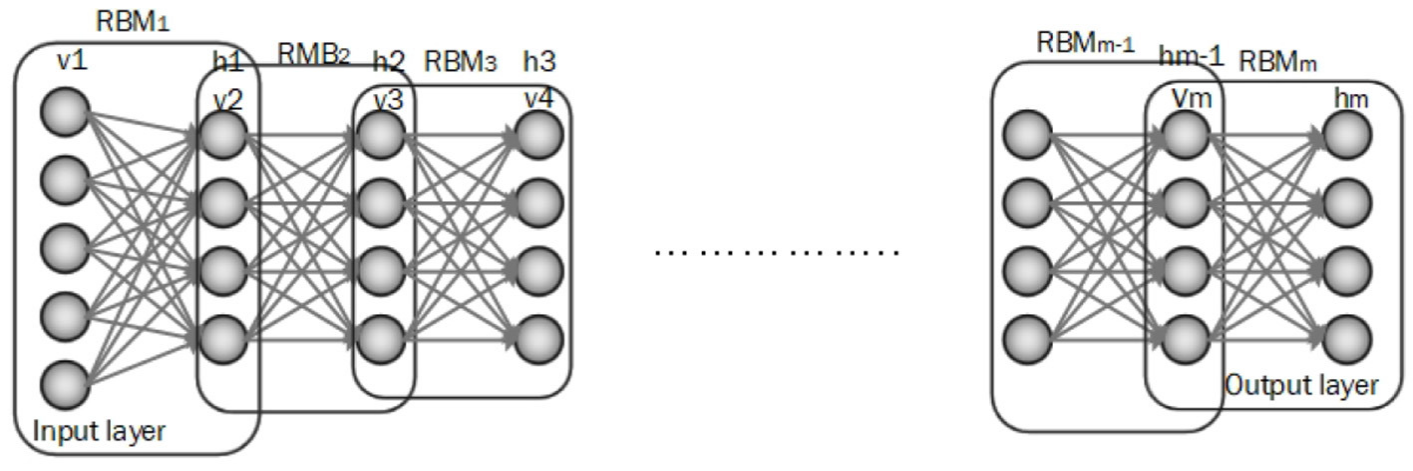
Illustration of a DBN architecture.

DBN is composed of multiple Boltzmann machines (RBMs) layers which are ordered subsequently. Each RBM includes a visible and hidden layer and is trained by the greedy learning algorithm to represent features. In DBN architecture input data is fed to the visible layer of the first RBM and then the hidden layer of the first RBM is an input of the next RBM and this process is continued until achieving a hidden layer of the final RBM. The top layer of DBN is the output layer (Irene et al., 2020; Hinton et al., 2006). An architecture for the DBN is shown in Figure 6.

## 4 Database

Most of the articles use existing online databases to train the proposed deep learning method and also test the performance of their PCG analysis system. The most common public databases used by the articles are:

Physionet PCG Dataset (PHSDB), The PASCAL Classifying Heart Sounds Challenge (CHSC2011), The Heart Sounds Shenzhen Database (HSSDB), The Michigan Heart Sound and Murmur database (MHSDB), The Massachusetts Institute of Technology heart sounds database (MITHSDB), Kaggle, UC Irvine Machine Learning Repository (UCI), UoC murmur, Cleveland, University of Washington, and Yaseen et al. (2018). These databases are listed in Table 2.

**Table 2:** List of databases.

**Database**	**Way to access**	**Size of database**
**PHSDB**	**http://physionet.org/content/challenge-2016/1.0.0/**	**The training set consists of five databases (A through E) containing a total of 3,126 heart sound recordings**
CHSC2011 Kaggle	http://www.peterjbentley.com/heartchallenge/	Dataset A containing 176 recordings: Normal, Murmur, Extra Heart Sound, Artifact. Dataset B contains 656 recordings: Normal, Murmur, and Extrasystole
MHSDB	https://open.umich.edu/find/open-educational-resources/medical/heart-sound-murmur-library	It consists of 23 heart sound recordings
HSSDB		Containing 845 recordings. The recordings were collected from patients with coronary heart disease, arrhythmia, valvular heart disease, congenital heart disease, etc.
MITHSDB	Can be found in PhysioNet 2016	Containing 409 recordings from 5 groups: normal, mitral valve prolapse (MVP), innocent or benign murmurs, aortic disease (AD), and other miscellaneous pathological conditions (MPC)
UoC murmur	This database is proprietary and is not publicly available	Containing 336 recordings from healthy children with innocent murmurs and various forms of CHD
The UC Irvine Machine Learning Repository	http://archive.ics.uci.edu/ml/index.php	Containing 622 data sets as a service to the machine learning community
Cleveland	Selected from the UCI machine learning repository	Containing 303 recordings
University of Washington	http://depts.washington.edu/physdx/heart/tech1.html	
Yaseen	http://github.com/yaseen21khan/Classification-of-Heart-Sound-Signal-Using-Multiple-Features	Containing 1,000 recordings from 5 classes AS, MR, MS, MVP, and Normal

## 5 Performance measures

A formulation for quantitatively evaluating the performance of a classifier, based on the outcomes of the validation, is known as the performance measure. The performance measures which are commonly seen in the related publications reflect an aspect of the classification performance. In this study, we face binary classification, where the result of the classification can be either normal or abnormal. The classifier output can be either positive or negative, relying on abnormal or normal conditions, respectively. The prediction value of a classifier can be either true or false, for the correct and incorrect classification, respectively. We may, therefore, face one of the following situations:

True Positive (TP): The model predicts the positive class correctly

True Negative (TN): The model predicts the negative class correctly

False Positive (FP): The model predicts the positive class incorrectly

False Negative (FN): The model predicts the negative class incorrectly

Based on these definitions, the following performance measures named: Accuracy, Sensitivity, Specificity, Recall, Precision, Positive Predictive Value (PPV), Negative Predictive Value (NPV), G-mean, and F1-Score are calculated as follows:


(1) $$\begin{array}{l} Accuracy=\frac{TP+TN}{TP+TN+FP+FN} \end{array}$$

Accuracy is a performance measure that reflects the ability of a classifier to segregate different classes. For example, in an abnormal-normal heart sound classification problem, the accuracy of a classifier shows how well the two classes are separated by the classifier. This performance measure considers the two classes equally.


(2) $$\begin{array}{l} Sensitivity=\frac{TP}{TP+FN}\;Specificity=\frac{TN}{TN+FP} \end{array}$$

The sensitivity of a classifier in the above example, is the performance measure that indicates the capability of the classifier in the correct classification of the abnormal class (low value of *FN*), while the sensitivity is the capability in the correct classification of the normal class (low *FP*).


(3) $$\begin{array}{l} Recall=\frac{TP}{TP+FN}\;Precision=\frac{TP}{TP+FP} \end{array}$$

The precision of a classifier indicates the capability of the classifier in the correct classification of the normal class concerning the normal labels assigned by the classifier.


(4) $$\begin{array}{l} PPV=\frac{TP}{TP+FP}\;NPV=\frac{TN}{TN+FN} \end{array}$$

(5) $$\begin{array}{l} F1-Score=2.\frac{Precision.Recall}{Precision+Recall} \end{array}$$

(6) $$\begin{array}{l} UAR=\frac{\sum_{i=1}^{N_{c}}Recall_{i}}{N_{c}},\left(N_{c}:thenumberofclasses\right) \end{array}$$

(7) $$\begin{array}{l} G-mean=\sqrt{Sen.Spe} \end{array}$$

The *F*1−*Score*, *NPV*, and *PPV* of a classifier, altogether indicate the capability of the classifier to provide a good balance between correct classification of the normal and abnormal classes. Unweighted Average Recall (UAR) and Geometric mean (G-mean) are used to evaluate model performance, especially when the learning dataset is imbalanced, such that a higher G-mean or UAR indicates that the model performs well on both classes of a binary case. These performance measures become important when a heavy class imbalance is seen in the learning database, as the class with the larger group size can overwhelm the minority class if accuracy is employed as the performance measure only.

## 6 Research methodology

We performed a topical survey retrospectively using the reachable reports, published in the technical, interdisciplinary, and medical journals or conference proceedings between 2017 and 2023. The research method is composed of 3 steps: search, screening, and eligibility.

### 6.1 Search

The three major search engines of the field are invoked to find the publications: PubMed, ScienceDirect, and Google Schola. The following keywords are employed as the keywords for the query:

- “Deep Learning” and (“Heart sound” or “Phonocardiogram” or “Phonocardiography”)
- “Convolutional Neural Network” and (“Heart sound” or “Phonocardiogram” or “Phonocardiography”)
- “Deep Machine Learning” and (“Heart sound” or “Phonocardiogram” or “Phonocardiography”)
- “Time Growing Neural Network” and (“Heart sound” or “Phonocardiogram” or “Phonocardiography”)

In this step, the title of the papers is explored to exclude repetitive and irrelevant records. The identified papers are passed to the next step for the screening.

### 6.2 Screening

The abstracts of the papers found in the Search are studied in terms of both the technical contents and the application. Those papers addressing irrelevant topics and the ones that are not accessible by the mentioned search engines are excluded from the study.

### 6.3 Eligibility

In this step, the papers passing through the previous two steps are explored in terms of the availability of the full paper. Next, the inclusion criteria are investigated followed by exploring the exclusion criteria. The papers, passing through the whole filters will be selected to be thoroughly studied.

#### 6.3.1 Inclusion criteria

- The central focus of the publications was the development or review of deep learning methods, applied to human heart sound signal.
- The publication dates lay between 2017 and 2023.

### 6.4 Exclusion criteria

Those publications which meet at least one of the exclusion criteria did not participate in the study:

- Incomplete reporting of the joint performance measures: either accuracy-sensitivity or accuracy-specificity (In biomedical studies, the balance between sensitivity and specificity is an important factor, reflecting the performance of the methods).
- Inaccessibility of the publication full-text.
- The paper is not a survey paper. The survey papers are studied for the discussiona.

## 7 Taxonomy of the survey

The articles found by the survey, tackle one of the following 6 research questions: feature extraction, classification, end-to-end learning (feature extraction + classification), and segmentation. Disease detection, disease classification, and severity assessment of cardiac disease are all considered as the applications of the study, fitting well into the classification category. Consequently, the survey taxonomy is based on the below-described research questions according to our findings. The results of the study will be presented in line with the taxonomy in the next section

### 7.1 Feature extraction problem

The extraction of concise and informative data content to improve segregation between different data groups is known as feature extraction. The effectiveness of the extracted should be validated considering dependencies over the feature space. This makes finding a feature set with an optimal discrimination power, problematic. Table 3 lists these papers. We observed that using deep learning techniques for feature extraction cannot noticeably improve the classification accuracy, unless dynamic contents of the features, or a fusion of the deep and the hand-crafted features are constructed.

**Table 3:** Categorizing DL networks based on the taxonomy of studied papers.

**DL method**	**Feature extraction**	**Classification**	**End-to-end learning**	**Segmentation**
CNN	Li H. et al., 2020; Demir et al., 2019; Humayun et al., 2018a, 2020; Zhang and Han, 2017; Alaskar et al., 2019; Bae et al., 2020; Colt et al., 2021	Asmare et al., 2020; Chen et al., 2020b; Dominguez-Morales et al., 2017; Maknickas and Maknickas, 2017; Bozkurt et al., 2018; Rubin et al., 2017; Kucharski et al., 2017; Wibawa et al., 2018; Khan et al., 2021; Li F. et al., 2020; Noman et al., 2019; Li et al., 2019; Banerjee and Majhi, 2020; Duggento et al., 2020; Singh et al., 2020; Dhar et al., 2021; Low and Choo, 2018; Chakraborty et al., 2020; Malik et al., 2020; Li et al., 2017; Kucharski et al., 2019; Kang et al., 2018; Han et al., 2018; Nogueira et al., 2019; Wu et al., 2019; Cheng et al., 2019; Ho et al., 2021; Tseng et al., 2021; Bilal, 2021; Rizal et al., 2020; Jeong et al., 2021; Hettiarachchi et al., 2021; Chowdhury M. et al., 2020; Lv et al., 2021; Tiwari et al., 2021; Takezaki and Kishida, 2021; Li T. et al., 2021; Huai et al., 2021; Kui et al., 2021; He et al., 2021; Tiwari et al., 2020; Alqudah et al., 2020; Koike et al., 2020; Banerjee and Ghose, 2020; Sundaram et al., 2021; Sugiyarto et al., 2021; Kesav et al., 2021; Xu and Lin, 2021; Jyothi and Pradeepini, 2021a; Duggento et al., 2021; Yang et al., 2021; Boulares et al., 2021	Krishnan et al., 2020; Baghel et al., 2020; Kiranyaz et al., 2019; Avanzato and Beritelli, 2020; Shojaedini and Morabbi, 2020; Xu et al., 2018; Kayikçı, 2019; Xiao et al., 2019; Gjoreski et al., 2020; Deperlioglu, 2019b; Chorba et al., 2021; Deperlioglu, 2018; Humayun et al., 2018b; Xiao et al., 2020; Joshi et al., 2020; Samir et al., 2021; Oh et al., 2020	Meintjes et al., 2018; Renna et al., 2019; Xu et al., 2020; Yin et al., 2020
RNN	Zhang et al., 2019	Latif et al., 2018; Naveen et al., 2021; Khan et al., 2020	Sujadevi et al., 2017	Chen et al., 2020a; Messner et al., 2018; Fernando et al., 2019; Xu et al., 2019; Oliveira et al., 2020
CNN-RNN	Li P. et al., 2021	Deng et al., 2020; Alam et al., 2018; Ali et al., 2020; Wang J.-K. et al., 2020; Shinde and Martinez-Ovando, 2021	Shuvo et al., 2021; Alkhodari and Fraiwan, 2021; Megalmani et al., 2021; Ali et al., 2021; Liu et al., 2022; Chen et al., 2022; Ren et al., 2022b; Al-Issa and Alqudah, 2022	Chen Y. et al., 2021
TGNN	Gharehbaghi and Lindén, 2017; Gharehbaghi et al., 2017a, 2018, 2019b,c,a,d, 2020, 2021	-	-	-
DBN	-	-	Irene et al., 2020	-
MLP	-	Chowdhury T. H. et al., 2020; Sotaquirá et al., 2018; Bondareva et al., 2021; Ghosh et al., 2020	Deperlioglu et al., 2020; Kose et al., 2021; Deperlioglu, 2021, 2019a	Mishra et al., 2018; Wang X. et al., 2020; Babu and Ramkumar, 2020

### 7.2 Classification problem

Likewise, DL methods have been dominantly used in many case studies to perform classification. In most of the cases of heart sound classification, discrimination between normal and abnormal heart is the study objective (Chen et al., 2020b; Dominguez-Morales et al., 2017; Bozkurt et al., 2018). However, the detection of a certain heart abnormality versus other heart abnormalities along and/or normal heart conditions is observed to be the main goal of some studies (Li H. et al., 2020; Wang J.-K. et al., 2020). Segregation between different classes of heart sound is also seen in a number of the studies on heart sound signal classification (Li et al., 2017; Kang et al., 2018; Boulares et al., 2021). Table 3 shows all these papers.

### 7.3 End-to-end learning

In the heart sound analysis domain, End-to-End learning implies studies in which feature extraction and classification are performed simultaneously. A considerable number of the reviewed papers used a DL method for end-to-end learning. Table 3 represents these papers.

### 7.4 Segmentation problem

In many studies, the heart sound signal is firstly pre-processed and the cardiac cycles as well as the first heart sound and the second heart sounds are fully localized on the heart sound recordings before the rest of the learning process. DL methods have been recently employed to perform this phase of the heart sound signal analysis by many researchers. Table 3 shows these papers.

## 8 Results

**Figure 7:**
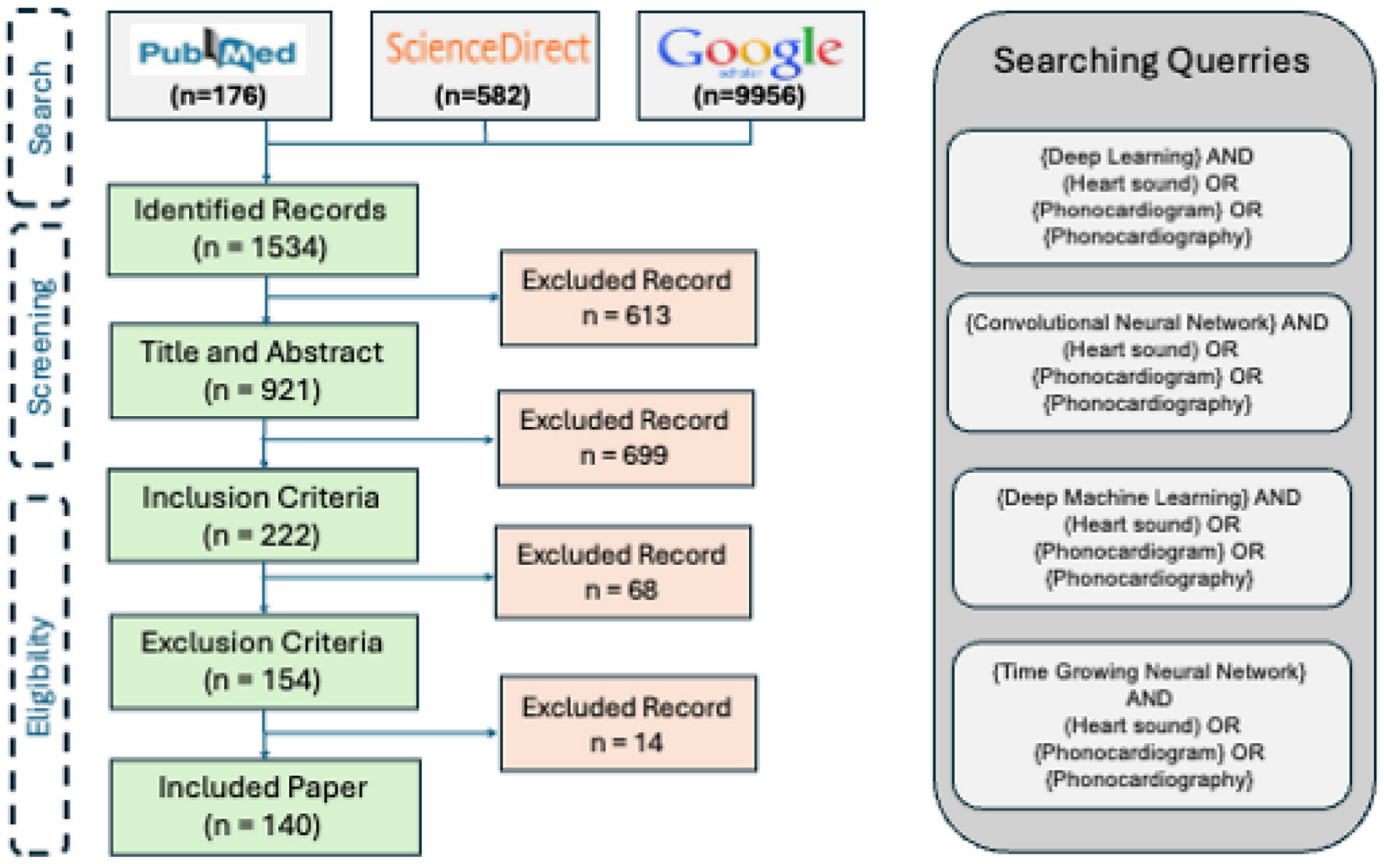
The Prisma graph of the survey findings.

**Figure 8:**
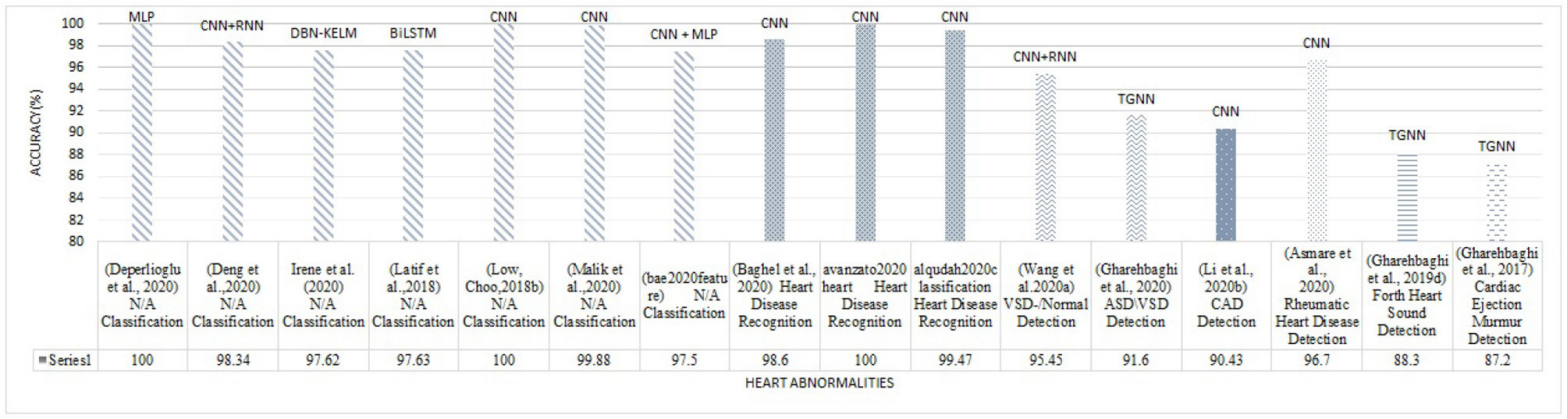
The best accuracy of each deep learning method for abnormalities classification (N/A: Normal/Abnormal).

Figure 7 illustrates the results of the research methodology as was described in Section 6. The query performed in the mentioned search engines resulted in 10,716 records where most of the records were either repetitive or irrelevant (see Section 6). The number of records was 1,534 from which 222 recordings were observed to be relevant according to the Title and the Abstract, and the ultimate number of the papers to be studied was 140 papers. In total, 14 survey articles were found, whose results will be compared and described in the discussion, Section 9. Figure 7 illustrates the findings of the survey. In order to provide an understanding of the methodological superiority in terms of the experimental results, the outperforming methods will be represented together with the detailed results, according to the study taxonomy. Figure 8 demonstrates the accuracy of the outperforming methods according to the taxonomy.

The superior performance was found in the studies by Mishra et al. (2018) on the classification problem, and by Deperlioglu et al. (2020) on the segmentation problem, and by Avanzato and Beritelli (2020) on the End-to-End learning. It is important to note that providing a realistic comparison of the accuracy for different learning methods requires clear information about the validation method employed for accuracy estimation. This is tightly linked to the training, validation, and testing databases and also the classification question. Group size, class similarities, corresponding to the cardiac disease conditions, and the data percentage used for training/validation/testing as well as the data selection manner all affect the estimated accuracy. Classification question is, yet, another important point affecting the accuracy, as an abnormal/normal case exhibits a different learning than a classification question in which a single vs. all classes is the objective. Nevertheless, this figure demonstrates appropriate pointers to the references where the validation process is detailed. Tables 4–8, also represent more details of the results found by this survey, including the segmentation manner. Results of the outperforming methods along with the implementation and the validation details are described in the following sequels according to the study taxonomy.

**Table 4:** A complete list of the CNN-based methods and their performance found by the study.

**Application**	**References**	**Database**	**Method**		**Segmentation**	**Performance**
			**Feature extraction**	**Classification**		
Rheumatic heart disease detection	Asmare et al., 2020	Collected data	Spectrogram	CNNs	-	Acc: 96.7%, Sen: 95.2%, Spe: 98.2%
Abnormal vs. Normal	Krishnan et al., 2020	PHSDB	1D-CNN		-	MAcc: 75.0%, Sen: 58.0%, Spe: 93.0%
Heart disease detection	Baghel et al., 2020	Collected data by Yaseen et al. (2018)	CNN		-	Acc: 98.6%
CAD detection	Li H. et al., 2020	Collected data	Fusion features: Multi-domain feature, (MFCC + CNN)	MLP	LR-HSMM	Acc: 90.43%, Sen: 93.67%, Spe: 83.36%, G-mean: 88.19%
Abnormal vs. normal	Chen et al., 2020b	PHSDB	Modified frequency slice wavelet transform	Combined of two CNN models	HMM	MAcc: 93.91%, Sen: 95.04%, Spe: 92.79%
Normal, murmur, extra heart sound, artifact	Demir et al., 2019	PASCAL-CHSC2011	Spectrogram-AlexNet + Spectrogram-VGG16 + Spectrogram-VGG19	SVM	-	Precision: normal: 59%, murmur: 77%, extra heart sound: 83%, artifact: 100%
Abnormal vs. Normal	Dominguez-Morales et al., 2017	PHSDB	Gray-scale sonogram image	Modified AlexNet	-	MAcc: 94.16%, Acc: 97.05%, Sen: 95.12%, Spe:93.20%
Abnormal vs. normal	Humayun et al., 2018a	PHSDB	CNN Model including a learnable front end filter bank	MLP	LR-HSMM	MAcc: 87.10%, Sen: 90.91%, Spe: 83.29%
Abnormal vs. normal	Bozkurt et al., 2018	UoC-murmur, PHSDB	Sub-band envelope	CNN	Period asynchronous segmentation	Acc: 81.5%, Sen: 84.5%, Spe: 78.5%
Abnormal vs. normal	Humayun et al., 2020	PHSDB	CNN	MLP	LR-HSMM	MAcc: 80.91%, Sen: 90.58%, Spe: 71.23%, F1: 81.96%
Abnormal vs. normal	Kiranyaz et al., 2019	PHSDB	1D-CNN trained with a novel data purification approach		Temporal beat segmentation	Sen: 89.67%, Spe: 86.89%, Ppr: 69.70%
Abnormal vs. normal	Rubin et al., 2017	PHSDB	MFCC	CNN	LR-HSMM	Sen: 72.78%, Spe: 95.21%, Overall: 83.99%
Abnormal vs. normal	Kucharski et al., 2017	The Aalborg University database from PHSDB	Spectrogram	CNN	-	Sen: 99.1%, Spe: 91.6%
Abnormal vs. normal	Alaskar et al., 2019	PHSDB	Scalogram + AlexNet	SVM	LR-HSMM	Acc: 87.65%, Sen: 83.71%, Spe: 89.99%, PPV: 83.22%, NPV: 90.30%, Positive likelihood: 8.36, Negative likelihood: 0.18
Abnormal vs. normal	Wibawa et al., 2018	PASCAL-CHSC2011	Spectrogram	CNN	-	Acc: 82.83%
Heart disease detection	Avanzato and Beritelli, 2020	Collected data by Yaseen et al. (2018)	CNN		-	Analysis window (2, 6): sec: Acc: (96.6%, 100%), Sen: (96.8%, 100%), Spe: (96.8%, 100%), F1-Score: (96.9%, 100%)
Abnormal vs. normal	Shojaedini and Morabbi, 2020	PASCAL-CHSC2011 + Kaggle	Spectrogram	CNN	-	Acc: in the range of 93%–98% when the FPR was 0% and in the range of 96%–96.5% when the TRP was 100%.
Abnormal vs. normal	Khan et al., 2021	PASCAL-CHSC2011	Spectrograms	CNN	-	Acc: 96.8%, Sen: 95.8%, Spe: 98%, Precision: 98.29%, F1-Score: 97.05%
Abnormal vs. normal	Li F. et al., 2020	PHSDB	Time and frequency domains, Skewness and Kurtosis, Cyclostationary domain, Entropy domain, Cepstrum	CNN	LR-HSMM	MAcc: 86.8%, Sen: 87%, Spe: 86.6%, MCC: 72.1%
Abnormal vs. normal	Noman et al., 2019	PHSDB	Raw (duration-normalized) + MFCC	Ensemble CNN	LR-HSMM	Acc: 89.22% Sen: 89.94% Spe: 86.35% MAcc: 88.15%
Abnormal vs. normal	Li et al., 2019	Collected data + PHSDB	Spectrogram + DAE (Denoising auto encoder	1D-CNN	-	Acc: 99.01%, F1-Score: 99.10%
Abnormal vs. normal	Banerjee and Majhi, 2020	PASCAL-CHSC2011	MFCC	CNN	-	Overall Acc: 83%
Abnormal vs. normal	Zhang and Han, 2017	PASCAL-CHSC2011	Spectrogram + CNN	SVM	-	Normalized total precision: 71%
Abnormal vs. normal	Low and Choo, 2018	MHSDB	Intensity map	CNN	Peak detection	Acc: 100%
Abnormal vs. normal	Xu et al., 2018	PHSDB	1-D CNN (a block stacked style architecture with clique blocks, and in each clique block a bidirectional connection)		-	Acc: 93.21%, Sen: 85.81%, Spe: 95.12%, Score: 90.46%
Abnormal vs. normal	Kayikçı, 2019	PASCAL-CHSC2011	CNN		-	Precision: Normal: 86%, Murmur: 68%, Recall: Normal: 46%, Murmur: 94%, F1-Score: Normal: 60%, Murmur: 79%
Abnormal vs. normal	Duggento et al., 2020	Collected data	MFCC	CNN + fully connected DNN	Duration-dependent HMM	F1-Score: Normal: 67%, Abnormal: 70%, AUC: 77%
Abnormal vs. normal	Singh et al., 2020	PHSDB	Scalogram	CNN	-	Acc: 87.96%, Sen: 88.58%, Spe: 87.80%
Abnormal vs. normal	Dhar et al., 2021	PHSDB	Cross-wavelet transform	AlexNet	-	Acc: 98%, Sen: 98%, Spe: 98%
Abnormal vs. normal	Xiao et al., 2019	Collected data	1D-CNN		-	Acc: 93.56%, Sen: 85.29%, Spe: 95.73%, Score: 90.51%
Abnormal vs. normal	Maknickas and Maknickas, 2017	PHSDB	MFSC	CNN	LR-HSMM	MAcc: 84.15%, Sen: 80.63%, Spe: 87.66%
Normal-abnormal	Gjoreski et al., 2020	PHSDB	Random forest (features) + Spectro-temporal ResNet (end-to-end)		-	Acc: 92.9%, Sen: 82.3%, Spe: 96.2%
Abnormal vs. normal	Chakraborty et al., 2020	PHSDB	Spectrogram	CNN	LR-HSMM	Score: 86.57%, Sen: 89.78%, Spe: 83.37%
Abnormal vs. normal	Malik et al., 2020	Open source collected data	Scalogram	CNN	-	Acc: 99.88%, Sen: 99.9%, Spe: 99.79%
Normal, murmur, extra heart sound, artifact	Li et al., 2017	PASCAL-CHSC2011	FFT	CNN	-	Precision: normal: 96%, murmur: 100%, extrasystole: 98%, artifact: 100%, total precision: 98%
Abnormal vs. normal	Deperlioglu, 2019b	PHSDB	CNN		The resampled energy method	Overall Score: 97.21%, Sen: 94.78%, Spe: 99.65%
Abnormal vs. normal	Chorba et al., 2021	Collected data	ResNet		-	Sen: 76.3%, Spe: 91.4%
Normal, murmur, extrasystole	Deperlioglu, 2018	PASCAL-CHSC2011	CNN		-	Acc: 97.9%, Sen: 99.47%, Spe: 98.42%
Abnormal vs. normal	Humayun et al., 2018b	HSSDB + PHSDB	Hierarchical with fusion		-	Acc: 64.2%, UAR: 42.1%
Abnormal vs. normal	Kucharski et al., 2019	An open access database	Spectrogram	CNN	-	Sen: 91%, PP: 99%, F1-Score: 94%
Normal, *S*_3_, *S*_4_, systolic & diastolic murmur	Kang et al., 2018	Open databases	Spectrogram	CNN	A robust method based on music beat tracking	Galaxy S5: Acc: 90%, Sen: 94%, Spe: 86%, PPV: 88%, NPV: 92%
Abnormal vs. normal	Xiao et al., 2020	PHSDB	CNN (a block-stacked style architecture with clique blocks, and in each clique block a bidirectional connection)		-	Acc: 93%, Sen: 86%, Spe: 95%, Score: 91%
Abnormal vs. normal	Han et al., 2018	PHSDB	MFCC	CNN	LR-HSMM	MAcc: 91.50%, Sen: 98.33%, Spe: 84.67%
Abnormal vs. normal	Nogueira et al., 2019	PHSDB	Eight features in time domain + MFCC	CNN	LR-HSMM	Overall Acc: 57.60%, Sen: 81.34%, Spe: 33.86%
Abnormal vs. normal	Wu et al., 2019	PHSDB	Spectrogram + Mel spectrogram + MFCC	Ensemble CNN	-	MAcc: 89.81%, Sen: 91.73%, Spe: 87.90%
Abnormal vs. normal	Cheng et al., 2019	PHSDB	Spectrogram	CNN	-	MAcc: 89.5%, Sen: 91%, Spe: 88%
Abnormal vs. normal	Ho et al., 2021	PHSDB	Frequency sub-band	CNN	-	Acc: 95.1%, Sen: 89.2%, Spe: 95.3%
Abnormal vs. normal	Tseng et al., 2021	PHSDB	Homomorphic, Hilbert, PSD envelope	Large kernel network boosting	ECG	MAcc: 92.48%, Sen: 96.34%, Spe: 86.62%
Normal-murmur-extrasystole	Boulares et al., 2021	PHSDB	MFCC	Fine-tuned VGG19	Unbiased autocorrelation function + Gaussian mixture model Bi-clustering	Acc: 97%, Sen: 94.6%, Spe: 94.6%
Abnormal vs. normal	Bilal, 2021	PHSDB	(1D-local binary pattern + 1D-local ternary pattern) Relief-based feature selection method	1D-CNN	-	Acc: 91.78%, Sen: 90.77%
Abnormal vs. normal	Rizal et al., 2020	PHSDB	Scalogram	ResNet-50	-	Sen: 93.4%, F1-Score: 93.3%, Precision: 93.7%
Abnormal vs. normal	Jeong et al., 2021	PHSDB	Spectrogram	CNN	-	Acc: 91%, Sen: 93.32%, Spe: 83%
Abnormal vs. normal	Hettiarachchi et al., 2021	PHSDB 2016, 2017	Scalogram	Hybrid CNN	-	Acc: 90.41%, Sen: 94.74%, G-mean: 84.29%
Abnormal vs. normal	Joshi et al., 2020	PASCAL-CHSC2011	CNN		-	Acc: 91.5%, Sen: 92%, Spe: 91%
Abnormal vs. normal	Chowdhury M. et al., 2020	PHSDB + MHSDB	MFCC + Mel-scaled power spectrogram	CNN	Shannon energy envelope	Overall Acc: 93.20%, Sen: 89.20%, Spe: 94.20%
Abnormal vs. normal	Lv et al., 2021	Collected data	Sub-band energies from the time-frequency images	CNN	-	Acc: 96%, Sen: 97%, Spe: 89%
Normal, murmur, extra systole, extrahls, artifact	Tiwari et al., 2021	PASCAL-CHSC2011	Spectrograms generated through Hybrid Constant-Q Transform	CNN	-	Acc: 96.4%, Recall: 96.6%, Precision: 93.4%, F1-Score: 94.8%
Abnormal vs. normal	Takezaki and Kishida, 2021	PHSDB	Spectrogram	CNN (ResNet18)	-	Acc: 92.5%, Sen: 86.3%, Spe: 94%
Abnormal vs. normal	Li T. et al., 2021	PHSDB	STFT	CNN	-	MAcc: 86%, Acc: 85%, Sen: 87%, Spe: 85%
Abnormal vs. normal	Huai et al., 2021	Collected data + PHSDB	Spectrogram	CNN	-	Acc: 94.8%, Sen: 94.29%, Spe: 95.54%, F1-Score: 93.84%, Precision: 93.44%, AUC: 94.3%
Abnormal vs. normal	Kui et al., 2021	Collected data	MFSC	CNN	Duration-dependent HMM	Acc: 93.89%, Sen: 92.78%, Spe: 95%
Abnormal vs. normal	He et al., 2021	PHSDB	Homomorphic, hilbert, wavelet and PSD envelope	CNN	U-net based on the deep CNN	Classification: Acc: 87.3%, Sen: 96.4%, Spe: 78.1% Segmentation: overall Acc: 99.1%
Normal-abnormal-artifact	Tiwari et al., 2020	PHSDB	MFCC based on proposed discrete cosine transform	CNN	-	Precision: 95%, F1-Score: 94%, Sen: 94%
Abnormal vs. normal	Bae et al., 2020	Collected data	Mel-spectrogram + pretrained inception V3 model	MLP	-	Acc: 97.5%
Abnormal vs. normal, heart disease detection	Alqudah et al., 2020	PHSDB	Bispectrum full images	CNN	-	Acc: 99.47%, Sen: 99.34%
Abnormal vs. normal	Koike et al., 2020	PHSDB	Log Mel spectrogram	Pretrained CNN (PANNs)	-	F1-Score: 79.1%, Sen: 96.9%, Spe: 88.6%, UAR: 89.7%
Abnormal vs. normal	Banerjee and Ghose, 2020	MHSDB	Spectrogram	Variational autoencoder based on CNN	-	MHSDB: Sen: 99%, Spe: 100%
Abnormal vs. normal	Colt et al., 2021	PASCAL-CHSC2011	1D-CNN encoder and WaveNet decoder	SVM	-	Sen: 40%, Spe: 96%
Abnormal vs. normal	Sundaram et al., 2021	PASCAL-CHSC2011	Spectrogram	CNN (AlexNet)	-	Acc: 77%
Normal, AP, CHF, HHD	Sugiyarto et al., 2021	Recorded data	Scalogram	CNN	-	Acc: 85%, Sen: 80%, Spe: 100%
Abnormal vs. normal	Samir et al., 2021	PHSDB + Kaggle	CNN-jSO (jSO is an optimization algorithm)		-	Acc: 94.12%
LVDD diagnosis	Yang et al., 2021	Collected data	STFT	CNN	LR-HSMM	Acc: 98.7%, Sen:98.6%, Spe: 98.8%
Abnormal vs. normal	Kesav et al., 2021	PHSDB	Spectrogram	CNN	-	Acc: 85%
Normal, abnormal, extrasystole	Xu and Lin, 2021	PASCAL-CHSC2011	MFCC	CNN	-	Acc: 82.23%
Abnormal vs. normal	Jyothi and Pradeepini, 2021a	Collected data	MFCC, Spectrogram	CNN	LR-HSMM	Acc: N: 98%, A: 85%
Abnormal vs. normal	Duggento et al., 2021	PHSDB	MFCC	Multi-branch CNN	Duration-dependent Hidden Markov Model	Acc: 74.6%, Sen: 97%
Heart disease detection	Oh et al., 2020	Yaseen et al., 2018	WaveNet (a generative model that consists of a residual block with gated activation. It is similar to the CNN model)		-	High Acc: 97%, Sen: 92.5%, Spe: 98.1%

Acc, Accuracy; Sen, Sensitivity; Spe, Specificity; MAcc, Mean accuracy; AUC, Area Under Curve; UAR, Unweighted Average Recall; LR-HSMM, Logistic regression Hidden Semi Markov Model; DNN, Deep Network; MFSC, Mel-Frequency Spectral Coefficients; PSD, Power Spectral Density; STFT, Short Time Fourier Transform.

**Table 5:** A complete list of the RNN-based methods and their performance found by the study.

**Application**	**References**	**Database**	**Method**		**Segmentation**	**Performance**
			**Feature extraction**	**Classification**		
Abnormal vs. normal	Zhang et al., 2019	PHSDB	Spectrogram + average magnitude difference function (AMDF) + LSTM	Two-layer neural network	-	Sen: 94.22%, Spe: 90.48%, Overall Score: 92.35%
Abnormal vs. normal	Latif et al., 2018	PHSDB	MFCC	Bi-LSTM	LR-HSMM	Acc: 97.63%, Sen: 98.86%, Spe: 98.36%
Abnormal vs. normal	Sujadevi et al., 2017	PASCAL-CHSC2011	LSTM		-	Acc: 76.9%, Precision: 83.3%, Recall: 76.9%, F-measure: 76.6%
Normal, murmur, artifact, extra systole	Naveen et al., 2021	Collected data	MFCC	LSTM	-	Acc: 94%, F1-Score: 94%, Recall: 93%, Precision: 94%
Abnormal vs. normal	Khan et al., 2020	PHSDB	MFCC	LSTM	-	Acc: 80.68%, Sen: 83.24%, Spe: 99.55%

**Table 6:** A complete list of the TGNN-based methods and their performance found by the study.

**Application**	**References**	**Database**	**Method**		**Segmentation**	**Performance**
			**Feature extraction**	**Classification**		
Normal, mild disease, critical disease	Gharehbaghi et al., 2019c	Collected data	TGNN	MLP	-	The estimated negative error: 0%, The positive error: 6.3%
ASD or VSD detection	Gharehbaghi et al., 2020	Collected data	TGNN	MLP	A method based on physiological effects of respiration	Acc: 88.4%, Sen: 91.6%, classification error: 9.89% using the A-Test method
Abnormal vs. normal	Gharehbaghi et al., 2019b	Collected data	Static TGNN + Moving TGNN + MLP	SVM	A method based on physiological effects of respiration	Acc: 84.2%, Sen: 82.8%, Spe: 85.7%, Classification error: 5.1%
Forth Heart Sound Detection	Gharehbaghi et al., 2019d	Collected data	Backward TGNN + MLP	SVM	ECG	Acc: 88.3%, Sen: 82.4%, Spe: 93.7%, classification error: 18.3% using the A-Test method
Stenosis and Regurgitation Murmurs Classification	Gharehbaghi et al., 2019a	Collected data	TGNN + MLP	SVM	ECG	Acc: 85%, Sen: 80%, classification error: 17.4% using the A-Test method
AS and BAV Classification	Gharehbaghi et al., 2021	Collected data	TGNN	MLP	ECG	Acc: 85.8%, classification error: 14.2% using the A-Test method
Cardiac Ejection Murmurs Detection	Gharehbaghi et al., 2017a	Collected data	A hybrid model and a TGNN	SVM	ECG	Confidence interval of: Acc: 87.2% - 88.8%, Sen: 83.4% - 86.9%, Spe: 88.3% - 90.0%
Abnormal vs. normal	Gharehbaghi and Lindén, 2017	Collected data	DTGNN	SVM	TGNN	Classification rate: 85.5%, Sen: 83.9%, Spe: 86%
Normal-PDA	Gharehbaghi et al., 2018	Collected data	DTGNN	MLP	ECG	Acc: 86%, Sen: 85%, Spe: 87%

**Table 7:** A complete list of the CNN&RNN-based methods and their performance found by the study.

**Application**	**References**	**Database**	**Method**		**Segmentation**	**Performance**
			**Feature extraction**	**Classification**		
Abnormal vs. normal	Deng et al., 2020	PHSDB	MFCC	CRNN	-	Acc: 98.34%, Sen: 98.66%, Spe: 98.01%
Abnormal vs. normal	Alam et al., 2018	MHSDB + PASCAL-CHSC2011 + PHSDB	Spectrogram + MFCC	Parallel combination of the RNN based BiLSTM & CNN	-	Sen: 96.09%, Spe: 100%, Score: 98.01%
Abnormal vs. normal	Ali et al., 2020	The Cleveland dataset	Linear discriminant analysis (LDA) + principal component analysis (PCA)	CNN + GRU	-	Acc: 94.5%, Recall: 95%, Precession: 94%, F1-Score: 94%, AUC: 94.5%
VSD-normal classification	Wang J.-K. et al., 2020	Collected data	STFT	TAP-CRNN	-	Acc: 95.45%, Sen: 97.18%, Spe: 91.98%, PPV: 96.15%, NPV: 94.15%
Heart disease detection	Shuvo et al., 2021	Collected by Yaseen et al. (2018)	CNN + Bi-LSTM		-	Acc: 99.6%
CVD detection	Li P. et al., 2021	PHSDB	CNN-LSTM-PCG and CNN-LSTM-ECG fused by Genetic algorithm	SVM	-	Acc: 87.3%, Sen: 90.3%, Spe: 84.5%
Heart disease detection	Alkhodari and Fraiwan, 2021	Washington + Texas + 3M + Michigan	CNN-BiLSTM		-	Acc: 99.32%, Sen: 98.30%, Spe: 99.58%
Abnormal vs. normal	Megalmani et al., 2021	PHSDB	Hybrid CNN and LSTM		-	Acc: 94.51%, Sen: 97.48%, F1-Score: 94.5%
Abnormal vs. normal	Ali et al., 2021	Cleveland	Hybrid CNN and LSTM		-	Acc: 93.7%
Abnormal vs. normal	Shinde and Martinez-Ovando, 2021	PASCAL-CHSC2011	MFCC + STFT	hybrid CNN and LSTM	-	Acc: 88.5%
ASD, VSD, PDA and combined CHD classification	Liu et al., 2022	Collected data	Residual convolution recurrent neural network (RCRnet)		-	Acc: 94%%–99.4%
Abnormal vs. normal	Chen et al., 2022	PHSDB	1D-CNN + LSTM		-	MAcc: 86%, Sen: 87%, Spe: 82%
Abnormal vs. normal, heart disease detection	Al-Issa and Alqudah, 2022	PHSDB, Yaseen et al. (2018)	CNN + LSTM		-	PHSDB: Acc: 93.77%, Sen: 99.63%, Spe: 92.42% Yaseen et al., 2018: Acc: 99.87(Augmented), Acc: 98.5%(non-Augmented)
Abnormal vs. normal	Ren et al., 2022b	PHSDB	Combining a 1D CNN-LSTM and a 2D CNN by channel attention mechanism		-	Acc: 97.15, Sen: 97.17, Spe: 97.13

**Table 8:** A complete list of the MLP-based methods and their performance found by the study.

**Application**	**References**	**Database**	**Method**		**Segmentation**	**Performance**
			**Feature extraction**	**Classification**		
Abnormal vs. normal	Deperlioglu et al., 2020	PASCAL-CHSC2011	AEN		-	Acc: 100%, Sen: 100%, Spe: 100%
Abnormal vs. normal	Chowdhury T. H. et al., 2020	PHSDB	Mel-scaled power spectrogram, MFCC	A 5-layer feed-forward DNN	Shannon energy envelope and zero crossing	Acc: 97.1%, Sen: 99.26%, Spe: 94.86%
Abnormal vs. normal	Sotaquirá et al., 2018	PHSDB	time, frequency and time-frequency	DNNs + the weighted probabilities	LR-HSMM	Overall Acc: 92.6%, Sen: 91.3%, Spe: 93.8%
Abnormal vs. normal	Kose et al., 2021	The Cleveland database	AEN		-	Acc: 99.13%, Sen: 97.90%, Spe:97.95%
Normal, murmur, extra systole	Deperlioglu, 2021	PASCAL-CHSC2011	Stacked autoencoder network		Resampled energy method	Acc: 99.8%, Sen: 100%, Spe: 100%
Abnormal vs. normal	Bondareva et al., 2021	PASCAL-CHSC2011	The INTERSPEECH ComParE 2018 feature set + Shannon energy based features	6 layer MLP	-	Precision on normal: 81%, Precision on murmur: 96%
Normal-extrasystole-murmur	Deperlioglu, 2019a	PASCAL-CHSC2011	Autoencoder neural network		resampled energy method	Acc:99.93%, Sen: 99.77%, Spe: 99.77%
Heart disease detection	Ghosh et al., 2020	Yaseen et al., 2018	L1-norm + sample entropy + and permutation entropy	Deep layer kernel sparse representation network (DLKSRN)	-	Acc: 99.24

### 8.1 Findings of the feature extraction problem

A number of the studies rely on using a DL method for feature extraction, in which the classification layer is independently trained for a certain study objective. The discrepancy in the methodologies and also in the study objectives, make the comparison problematic (see Table 3). Some papers use pre-trained networks to extract features, while others consider the classifier to be fixed and extract various features to evaluate its effect on classification problems. Thus the accuracy of feature extraction is reported as a measure. The DL methods, CNN, RNN, CNN-RNN, and TGNN, were differently employed by 19 studies, for feature extraction. Various types of CNN models are dominantly observed in these studies. As shown in Figure 8, a CNN model was reported to improve abnormal vs normal classification accuracy up to 97.5%, where the CNN was employed to extract discriminative features from mel-spectrum two-dimensional graphs (Bae et al., 2020). The ultimate classification was performed using an artificial neural network. In another study, a CNN model was employed for extracting powerful features from the colored images, resulting from applying the Mel-Frequency Cepstrum Coefficients (MFCC) to PCG signals.

Detection of coronary artery diseases was the study objective, and the accuracy was estimated to be 90.43% when a Multi-Layer Perceptron (MLP) neural network was used for the classification. Performance of the method was evaluated 5-fold using a dataset of PCGs collected at the Shandong Provincial Qianfoshan Hospital (see Table 4) (Li H. et al., 2020). TGNN served as a powerful method for feature extraction in different studies of PCG signal analysis. In all the TGNN-based methods, the spectral features, obtained by periodogram, were learned using different schemes of the growing windows, i.e., forward, backward, and bilateral growing windows, along with the discriminant analysis methods such as Fisher criteria and k-means clustering. The main objective of the studies was anomaly detection using either a support vector machine (SVM) or MLP for binary classification. Several case studies including, ASD vs. VSD, AS vs. BAV, and fourth heart sound detection were performed based on this combination, and accuracy of 88.4%, 85.8%, and 88.3% was reported, respectively (Gharehbaghi et al., 2020, 2021, 2019d) (see Table 6 for more details). In another study, a hybrid model composed of TGNN and hidden Markov Model was proposed for extracting indicative features of PCG signal in light of detecting cardiac ejection murmur, and the accuracy, sensitivity, and specificity were estimated to be 88.1%, 85.1%, and 89.2%, respectively (Gharehbaghi et al., 2017a).

### 8.2 Findings of the classification problem

**Figure 9:**
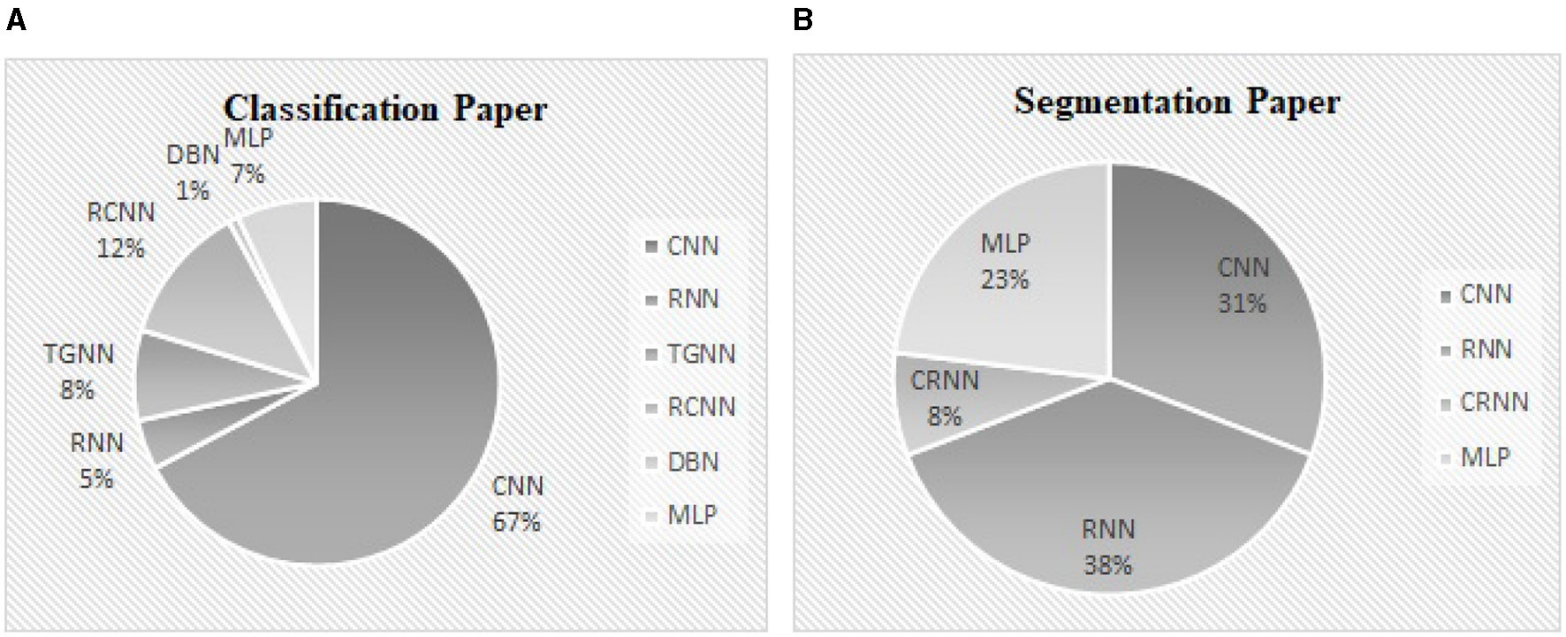
**(A)** The contribution percentage of different DL methods in the classification papers. **(B)** The contribution percentage of different DL methods in the segmentation papers.

Classification of abnormal versus normal heart conditions is the major research question tackled by various DL methods (Table 3). A total of the 63 papers employed DL methods for the classification, in which different architectures of CNN, RNN, RCNN, and MLP are observed. CNN-based methods are dominantly seen in the majority of the papers, contributing in 67% of the studies (see Figure 9A), where a perfect 100% accuracy was reported in one of the studies (Low and Choo, 2018). Figure 9A demonstrates methodological frequencies for the classification question. Although validation inconsistencies are seen in the validation methods and database, which make a fair comparison questionable, the versatility of CNN in this research question is conclusive. Figure 8 shows the best performance of each method. The methods with the superior accuracy for the underlying case studies are described in this sequel, and a complete list of all the studies together with the technical details and the study objectives are separately tabulated for each method in Tables 4–8.

A CNN with 2 convolutional layers, 2 max-pooling layers, and a kernel size of 11 × 11 was employed to classify abnormal PCGs from normal ones, using the intensity images obtained from the segmented PCG energies as the inputs. A perfect 100% accuracy was achieved by when the cross-validation with 80% 20% of training/test data was employed (Low and Choo, 2018). They used the MHSDB dataset for the validation and a dropout layer to avoid overfitting. As described in Table 4 and Figure 8, another study also reported a high accuracy of 98.7% for the CNN model when the bispectrum images of PCGs were employed as the input features (Alqudah et al., 2020). Classification of 5 heart diseases was the study objective. Their method was validated using a 10-fold method in conjunction with a dataset provided by Yaseen et al. (2018).

A combination of CNN and RNN was also proposed for the classification problem and results were compared to a parallel structure of RNN and CNN, named PRCNN, using MFCC as the input data (Deng et al., 2020). Proposed a method based on CRNNs for abnormalities classification. Accuracy, Recall, Precision, and F1-Score of the CRNN and PRCNN were calculated. As shown in Figure 8, the accuracy of the two methods was estimated to be 98.34% and 97.34%, respectively.

Temporal Attentive Pooling (TAP) was proposed for classifying the systolic murmur caused by VSD from the normal PCG (Wang J.-K. et al., 2020). Spectral features of PCGs were calculated using short-time Fourier transformation, and employed by the convolutional layers of a CNN-based architecture. The method architecture incorporated recurrent layers along with the TAP layers to learn the long-term dependencies of the convolutional layers. The classification was performed by the dense layers as the final layer. An accuracy of 95.45% was achieved when the 4-fold validation along with a dataset of PCGs prepared at the National Taiwan University Hospital was employed for the evaluation showing a performance improvement as compared to the CNN and the Convolutional Recurrent Neural Network (CRNN).

In another study, a CNN-based model was proposed using rheumatic heart disease as the case study for the classification (Asmare et al., 2020). Each PCG was divided into several temporal frames with a fixed length of 1.2 second. The mel-spectral contents were employed in their logarithmic form as the input features for a CNN with 5 convolutional layers and linear activation function. The method accuracy was estimated to be 96.7% using 80% 20% of training/test split of the dataset as shown in Table 4. The database for the evaluation was prepared at the Tikur Anbessa Referral Teaching Hospital College of Health Sciences, Addis Ababa University.

### 8.3 Findings of the end-to-end learning

End-to-End learning, implying learning heart sounds without performing the segmentation process, was found in 31 of the studies. Methods including, CNN, RNN, CNN-RNN, DBN, and MLP, were used to this end.

The best accuracy was obtained by an Auto Encoder Network (AEN) in two classification problems: a case with three classes, normal, murmur, and extra-systoles, and a case with two classes of normal and abnormal (Deperlioglu et al., 2020). An accuracy of 100% and 99.8% in the former and the later case, respectively when 80% 20% of training/test split was used for the validation, outperforming other methods such as ANNs, SVM, CNN, and DNN. Detail of the results can be found in Table 8.

An accuracy of 100% was also seen in another study for heart disease classification, in which CNN was employed for the learning process along with the recurrence filter with the temporal frames of 6 and 34 second (Avanzato and Beritelli, 2020). Cross-validation with 70%/30% of training/test data was employed in conjunction with the dataset in Yaseen et al. (2018).

Deep Belief Network was also employed through a hybrid method of fuzzy classifier, for the disease classification (Irene et al., 2020). Performance of the model was estimated using 10-fold validation, independently applied to three different datasets: Hungary dataset, Swiss dataset, and Cleveland dataset, and the accuracy was found to be 97.56%, 97.21%, and 97.62%, respectively.

### 8.4 Findings of the segmentation problem

Segmentation is by far less pronounced in the related studies on DL for PCG signals, observed in 15 of the studies. Different DL methods such as CNN, RNN, CNN-RNN and MLP were proposed for this research question, from which RNN and CNN are dominantly seen in the reports. As can be seen in Figure 9B, the DL methods which invoked RNN and CNN, contributed to 38%, 31% of the studies. The performance of the segmentation is evaluated by considering a window of 40 or 60 ms, where the predicted position of each heart sound must fall within this window to be correctly predicted. For example, a true positive is counted when the center of an S1 (S2) which occurs within the predicted label is closer than 40 ms to the center of the corresponding S1 (S2) in the ground truth label. Although metrics such as sensitivity and positive predictive have been used in some papers to evaluate segmentation performance, accuracy has been reported more often. Thus, we presented and compared the results based on their accuracy, even though it might sound inappropriate. However, there were 2 different DL methods that showed superiority over the rest of the methods found by the survey (Mishra et al., 2018). One of the methods was based on the one-dimensional CNN for the feature extraction, in conjunction with an MLP for S1-S2 classification. The other method employs a stacked auto-encoder for the classification which uses Mel-frequency cepstral coefficients as well as the related derivative as the input features. An accuracy of 100% and 99.8% was achieved by the two methods, respectively. Repeated random sub-sampling was used for the validation, and the three public datasets from the University of Washington, the University of Michigan, and Litman were invoked for the validation. Development and validation details of the DL methods for the segmentation found by the survey are included in Table 9.

**Table 9:** Results of deep learning methods for segmentation.

**Application**	**References**	**Database**	**Method**		**Performance**
			**Feature extraction**	**Classification**	
S1 and S2 classification	Meintjes et al., 2018	PHSDB	Scalograms	CNN	Acc: 86%, Sen: 87.4%, Spe: 86.7%, AUC: 93.8%
Heart sound segmentation	Renna et al., 2019	PHSDB	The homomorphic, Hilbert, wavelet and PSD envelope	CNN + HMM	Acc: 93.7%, Sen: 95.7%, PPV: 95.7%
Heart sound segmentation	Chen et al., 2020a	MITHSDB	The homomorphic, Hilbert, Wavelet and PSD envelope	BiLSTM	Sen: 96.36%, PPV: 95.88%, F1-Score (S1): 96.28%, F1-Score (S2): 95.98%, F1-Score: 96.11%
Heart sound segmentation	Messner et al., 2018	PHSDB	Spectrogram, MFCC, envelope features: homomorphic envelope, Hilbert envelope, wavelet envelope and PSD	BiGRNN	Sen: 95.9%, PPV: 94.9%, F1-Score: 95.4%
S1 and S2 classification	Mishra et al., 2018	Online data	1D-CNN	MLP	Overall mean Acc: 99.8%
Heart sound segmentation	Fernando et al. (2019)	M3-Hu	MFCC	Bi-LSTM with attention	Acc: 97.1%, Sen: 96.7%, Spe: 96.7%, F1-Score: 94.7%, PPV: 93.1%
Heart sound segmentation	Xu et al., 2019	PHSDB + the UCI database + University of Washington dataset + medical online + collected data	Simpler minimum gated unit (SMGU)-RNN		Acc: 88.56%
Heart sound segmentation	Oliveira et al., 2020	PHSDB	The homomorphic, Hilbert, wavelet and PSD envelope	Bi-LSTM	Sen increases 2.4% when compared to the standard approaches
S1 and S2 classifications	Xu et al., 2020	PHSDB	Personalized GMM-DHMM + MFCC	CNN	The final segmentation Acc: 92.92%
Heart sound segmentation	Chen Y. et al., 2021	MITHSDB	Convolutional long short-term memory (CLSTM)		F1-Score: 96.18%
Heart sound segmentation	Yin et al., 2020	MITHSDB	The homomorphic, Hilbert, wavelet and PSD envelope + STFT + 1D-CNN	Temporal convolutional network combined with Viterbi algorithm	F-Score: 97.02%
Heart sound segmentation	Wang X. et al., 2020	PHSDB	The temporal-framing adaptive network with an encoder-decoder architecture		Overall F1-score: 99.21%
S1 and S2 classification	Babu and Ramkumar, 2020	Real time data + PHSDB	The auto-correlation, Hilbert, homomorphic and PSD envelope extracted from empirical wavelet transform	U-Net based DNN	Acc: 91.17%

## 9 Discussion

**Figure 10:**
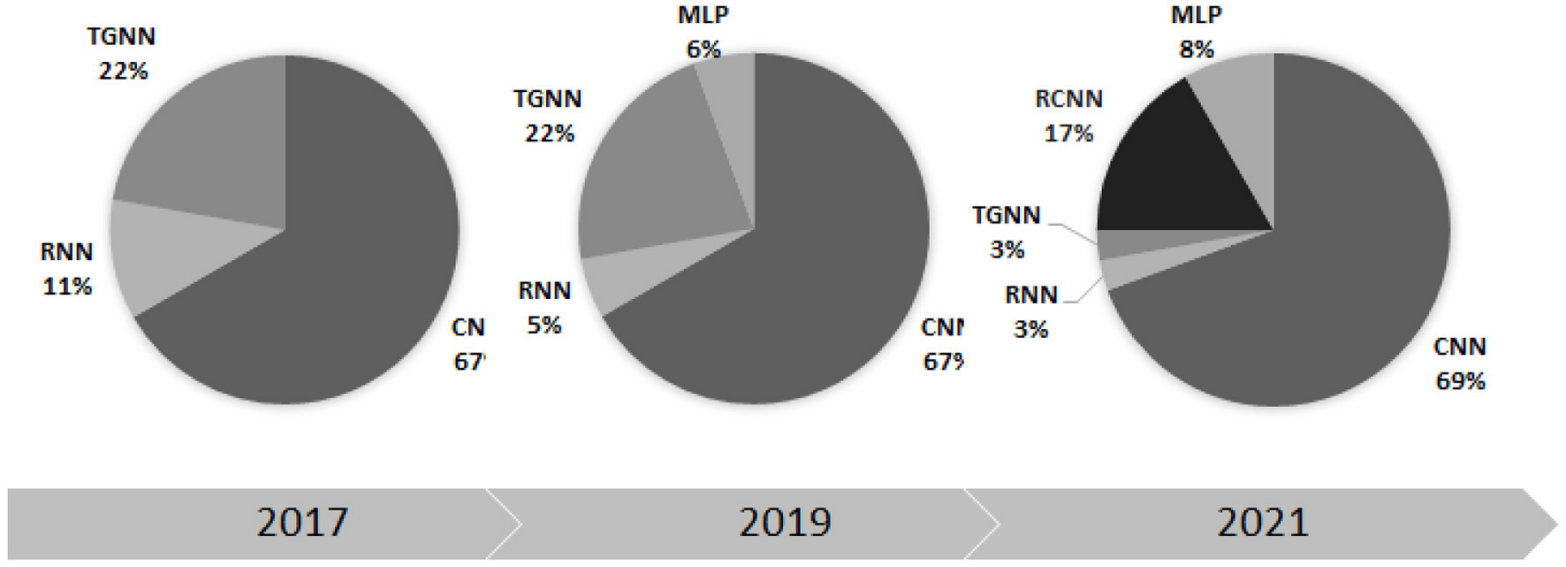
The percentage contribution of different deep learning methods in heart sound analysis for three recent years.

This paper considered all the scientific papers published in the well-used search engines within 2017–2023 and represented the results in the taxonomic order. Tables 4–8, listed all the methods along with the implementation details such as segmentation manner as well as validation database. The trend of the DL methodologies shows a shift toward further use of CNN for various applications of heart sound analysis (see Figure 10). It is seen that the total number of papers on heart sound analysis which were published within 2020–2021, is more than double the ones published within 2017–2020, showing a strictly positive trend of the research interest in this topic. This necessitates the need for a comprehensive survey study, to avail the researchers of the technical details along with the scope of the experimental results. A perfect 100% of the classification accuracy was observed in some of the DL-based studies (Deperlioglu et al., 2020; Low and Choo, 2018), showing a noticeable enhancement compared to the conventional methods (Rajamhoana et al., 2018). Nevertheless, strict conclusions about the performance of DL methods in this context demand further discussion, especially since the previous review papers fail to scrutinize the observed studies comprehensively.

A fair conclusion about the appropriate DL method for a research question demands some considerations beyond the performance measures. The method complexity including the segmentation and feature extraction methods are two key points requiring further attention when it comes to DL comparison. For a research question, the validation database in conjunction with the validation method should be considered in addition to the performance measures while several DL methods are to be compared.

### 9.1 Comparison to the other surveys

In total, 14 review or survey papers have been found in this study. A review paper on DL methods for PCG classification was the objective of one of the papers (Chen W. et al., 2021), which failed to consider a pervasive taxonomic and methodological study. Moreover, a number of the important CNN-based studies with high accuracy, were not addressed, making the methodological comparison crippled. The lack of objectivity and comprehensiveness of the study are seen in the two other review papers (Li S. et al., 2020; Rath et al., 2021b). Other studies, either addressed the classification problems only (Rajamhoana et al., 2018; Brites et al., 2021; Rath et al., 2021a; Vasantrao and Rangasamy, 2021; Fernando et al., 2021), or dealt with a narrow scope of the field (El-Dahshan et al., 2021; Jyothi and Pradeepini, 2021b). Oppositely, other review studies put different applications of DL methods into a broad scope of health informatics and rendered the general results without providing sufficient details of the learning and the validation process for PCG analysis (Abdullah Aloyuni, 2021; Bizopoulos and Koutsouris, 2018; Amin et al., 2021). Although heart disease detection was regarded as a narrow application of the DL method in some of the review studies, technical details of the learning and validation along with the results of the important papers with superior performance were overlooked (Lakshmi et al., 2021; Vasantrao and Rangasamy, 2021).

To the best of our knowledge, this study uniquely provides a pervasive knowledge about the state-of-the-art of DL methods along with the corresponding results in PCG analysis, including heart abnormalities classification, PCG segmentation, and recovery. In addition to the methodological and taxonomic contents, technical details of the validation methods, such as the PCG databases, were consistently rendered for each method.

### 9.2 Methodological complexity

One of the negative aspects of DL methods in comparison to conventional machine learning methods, is their high methodological complexity. It mainly refers to the computational power as well as the memory, required for learning and testing. The reviewed studies failed to report the complexity of their methods in a consistent manner. Nevertheless, a comparative study was found in which the training time of a simpler minimum gated unit (SMGU), MGU, LSTM, CNN, autoencoder, and RNN was estimated to be 2,395, 2,436, 2,863, 10,583, 1,186, and 1,839 seconds, respectively (Xu et al., 2019). It was also observed that for the classification problem, a CNN can demand more than 6 h time for a 10-fold validation using an Intel Core-I7 PC with 16 GB of memory when the bispectrum images employed as the inputs (Alqudah et al., 2020). In another study, the average training and testing time of a CRNN model that employed the MFCC input features was reported to be 3 h and 2.5 seconds, respectively, on a PC with a 3.5 GHz Intel core i5 CPU and 8 GB memory (Deng et al., 2020).

### 9.3 Performance comparison

Providing a realistic comparison of the performance measures over the reviewed papers is a big challenge due to the inconsistent validation process in terms of the method and the database. For example, in the classification problem, different values for the accuracy were reported, even as high as 100%, were reported (Low and Choo, 2018; Malik et al., 2020; Latif et al., 2018) (see Figure 8). One of the studies which reported a perfect 100% of accuracy, performs the validation by using repeated random sub-sampling and a database of 23 subjects only (Low and Choo, 2018), while another study with 99.88% of accuracy did so, using 5-fold validation method and a database of 1, 000 subjects with 5 different classes of PCGs (Malik et al., 2020). In terms of reliability, the latter is obviously preferred even though the accuracy is slightly degraded. Such validation discrepancy was observed in two other studies with the same objective, VSD detection, but the methodological difference: one employed a TGNN (Gharehbaghi et al., 2020) and the other one used a CNN-based method (Wang J.-K. et al., 2020). The accuracy of the TGNN and the CNN was estimated to be 88.4% and 97.1%, respectively. However, the reliability of the former is privileged due to the realistic validation process which employed repeated random sub-sampling method using a dataset of 115 subjects with 6 classes of PCG, whereas the latter one which employed an overlapping 2-fold validation (unclear overlapping manner) using a dataset of 150 subjects with only 2 distinct classes. Another computational aspect, that can direct the performance accuracy, is the segmentation manner employed for the classification. Some of the studies, particularly one that yielded a very high accuracy of 97.63% (Latif et al., 2018), ignored to report of the segmentation method, making the method reproducibility questionable.

### 9.4 Other methods

In addition to the above-described methods, a minority of other DL methods were found for different applications of PCG analysis. Bidirectional RNN and LSTM have been reported in a study for the classification task using the Physionet dataset, however, a significant improvement couldn't be found compared to the CNN (Sujadevi et al., 2019). Sharma and Dhar (2019) examined various deep learning techniques to classify heart sounds into normal, abnormal, and artifact. A combination of LSTM and CNN showed an improvement in the classification problem compared to the CNN (Netto and Abraham, 2021). It is also found that a deep TGNN can outperform the conventional hidden Markov model (Gharehbaghi and Babic, 2018).

One of the reviewed papers proposed a CNN-based method using ECG and PCG signals, for a classification problem with 4 classes: normal, abnormal, others, and noisy (Balbin et al., 2021).

Recent studies employed the attention mechanism to improve the performance of a CNN and RNN, and an enhancement in the performance was observed (Ren et al., 2022a). In some other studies, various combinations of CNN and Bidirectional LSTM with an attention block, have been proposed for the classification problem (Tian et al., 2022; Frimpong et al., 2022), as well as for the segmentation problem (Monteiro et al., 2022; Guo et al., 2022). However, these studies failed to meet the criteria for participation in the study.

## 10 Conclusions

This paper presented the results of a pervasive survey on deep learning methods for heart sound analysis, the topic that has recently received special interest from researchers. The reviewed papers were mainly focused either on disease classification from the segmented heart sound signals or on the methodologies for heart sound segmentation. To a lesser extent, applications such as end-to-end learning, heart sound recovery, and denoising were also observed. Among the different deep learning methods, the CNN-based method was dominantly seen in the classification problems, where a very high accuracy was reported by several studies. For the segmentation problem, the majority of the studies employed either a CNN-based or an RNN-based method. Although the complexity of CNN-based methods was by far higher than the RNN-based ones, the privileges of CNN in this context are conclusive. Regardless of the methodological complexities, much attention should be paid both to the validation method and to the learning database.
